# Agentic Search Engine for Real-Time Internet of Things Data †

**DOI:** 10.3390/s25195995

**Published:** 2025-09-28

**Authors:** Abdelrahman Elewah, Khalid Elgazzar, Said Elnaffar

**Affiliations:** 1 IoT Research Lab, The Department of ECSE, Ontario Tech University, Oshawa, ON L1G 0C5, Canada; abdelrahman.elewah@ontariotechu.ca; 2 Electrical Engineering Department, Benha Faculty of Engineering, Benha University, Benha 13511, Egypt; 3 School of Engineering, Applied Science and Technology, Canadian University Dubai, Dubai P.O. Box 117781, United Arab Emirates; said.elnaffar@gmail.com

**Keywords:** SensorsConnect, Agentic AI, LLM, RAG, ThingsDriver, IoT, C-IoT, IoT search engine

## Abstract

The Internet of Things (IoT) has enabled a vast network of devices to communicate over the Internet. However, the fragmentation of IoT systems continues to hinder seamless data sharing and coordinated management across platforms.However, there is currently no actual search engine for IoT data. Existing IoT search engines are considered device discovery tools, providing only metadata about devices rather than enabling access to IoT application data. While efforts such as IoTCrawler have striven to support IoT application data, they have largely failed due to the fragmentation of IoT systems and the heterogeneity of IoT data.To address this, we recently introduced SensorsConnect—a unified framework designed to facilitate interoperable content and sensor data sharing among collaborative IoT systems, inspired by how the World Wide Web (WWW) enabled shared and accessible information spaces for humans. This paper presents the IoT Agentic Search Engine (IoTASE), a real-time semantic search engine tailored specifically for IoT environments. IoTASE leverages LLMs and Retrieval-Augmented Generation (RAG) techniques to address the challenges of navigating and searching vast, heterogeneous streams of real-time IoT data. This approach enables the system to process complex natural language queries and return accurate, contextually relevant results in real time. To evaluate its effectiveness, we implemented a hypothetical deployment in the Toronto region, simulating a realistic urban environment using a dataset composed of 500 services and over 37,000 IoT-like data entries. Our evaluation shows that IoT-ASE achieved 92% accuracy in retrieving intent-aligned services and consistently generated concise, relevant, and preference-aware responses, outperforming generalized outputs produced by systems such as Gemini. These results underscore the potential of IoT-ASE to make real-time IoT data both accessible and actionable, supporting intelligent decision-making across diverse application domains.

## 1. Introduction

The Internet of Things (IoT) has reshaped human life through the growing number of connected things. Several domains have leveraged the implementation of the IoT, from home appliances and wearable devices to intelligent transportation and logistics systems. By the end of 2025, 75% of devices [1] will be connected to the internet. IoT devices are the nodes between the physical and digital domains. Conceptually, IoT devices [2] act as data sinks, receiving data to control connected actuators or data sources, sending data collected from embedded sensors. Therefore, they have become the primary real-time data sources and drains that represent a paradigm shift for real-time decision-making systems. Based on the statistics [1], the data generated or consumed by IoT devices will grow exponentially in the coming years. As a result, the demand for a real-time search engine for the collected IoT data will increase to keep pace with the needs of real-time decision-making.

Multiple attempts [3,4,5] in the literature endeavored to provide a search engine for IoT data. Shodan [6,7], a search engine for devices connected to the internet, crawls and indexes publicly available metadata of internetconnected devices. Shodan indexes data of a connected device, such as the type of the device, software used, the name and type of the deployed server and its version using devices’ banners. Thus, Shodan serves as a search engine to collect statistics [8] on connected devices in a specific region or gain insight into which software products are trending for market research purposes. Like Shodan, Censys [9] and Reposify _acquired by CrowdStrik [10] use crawling over the metadata of the connected devices to build their search engines. In contrast, these search engines focus on the security aspect of the connected devices. Organizations and enterprises can use these search engines to gain insight, such as determining vulnerable devices, assessing potential risks, and monitoring and scanning assets connected to the Internet. Thus, Shodan, Censys, or Reposify can retrieve only publicly available metadata of IoT devices, and we consider these types of search engines as device discovery tools. Based on the adopted approach (crawling over devices’ metadata), if these search engines try to extend their service to leverage the application data driven by connected devices, they will not access it due to the implemented authentication protocol restricting unauthorized connectors. Furthermore, assuming they gain the authority to access the application data, the fragmentation of the IoT devices and the heterogeneity of their data model pose further challenges.

IoTCrawler [11] is a framework designed to facilitate the search for data generated by IoT applications. It employs algorithms commonly used in web search engines, such as crawling, indexing, ranking, and discovery. By integrating IoTStream [12], which functions as a semantic annotation layer, IoTCrawler aims to enable effective search capabilities for existing IoT systems and to address the challenge of heterogeneity and fragmentation [13] within these systems.

The IoTCrawler group has made significant efforts to enable the search and discovery of IoT data; however, the framework has not seen widespread adoption within the IoT community. Even if IoTCrawler were to support all major IoT protocols–such as Message Queuing Telemetry Transport (MQTT), Constrained Application Protocol (CoAP), Hypertext Transfer Protocol/Secure (HTTP/HTTPS), and Advanced Message Queuing Protocol (AMQP)—the data formats and payload structures transmitted over these protocols remain highly inconsistent across different IoT systems. As a result, integrating the sensing data of a specific IoT system into IoTCrawler necessitates the development of a dedicated interface for each system. In other words, enabling access to sensing data through IoTCrawler would require managing an unmanageable number of custom Application Programming Interfaces (APIs), rendering the approach largely infeasible.

We recently introduced SensorsConnect [14], a framework that mirrors the architecture of the World Wide Web (WWW), but for IoT devices. As such, the use of a unified transfer protocol and messaging standard—similar to HTTP and HTML in the WWW—is applicable and beneficial. Two case studies [14], involving drive-through coffee shops and the USCanada border, demonstrated that leveraging a search engine for real-time IoT data in service recommendation applications reduced average service time by 46% and 31%, respectively.

Just as the exponential growth of websites on the WWW [15] presents a search challenge—due to the unstructured nature of web content—search engines [15] rely on hyperlinks for crawling, indexing, and ranking information. Likewise, with an estimated 41.6 billion IoT devices expected to generate 80 zettabytes of data by 2025, SensorsConnect will confront similar scalability and searchability challenges.

To address this, SensorsConnect is designed with native support for search functionality, thereby eliminating the need to implement traditional crawling, indexing, and ranking algorithms. Instead, it enables the integration of cutting-edge search technologies that have recently emerged for the WWW and are expected to replace traditional methods [16]. Focusing on its search engine component, this paper introduces a real-time IoT Agentic Search Engine (IoT-ASE), which leverages LLMs [17] and Retrieval-Augmented Generation (RAG) [18] approaches.

The remainder of this paper is organized as follows. Section 2 provides a brief overview of related work. Section 3 outlines the architecture of the IoT Retrieval-Augmented Generation Search Engine (IoT-RAG-SE) and introduces the Generic Agentic RAG (GA-RAG) workflow for agentic RAG systems. This section also details the implementation of IoTASE based on GA-RAG. Section 4 presents the evaluation of IoT-ASE using a real-world case study and discusses the corresponding experimental results. Finally, Section 6 offers concluding remarks.

## 2. Background and Related Work

### 2.1. SensorsConnect

A closer examination of the core challenge hindering the development of IoT search engines that support application-level IoT data reveals two primary causes: the fragmentation of IoT systems and the heterogeneity of sensing data. By design, many IoT systems have adopted intranet-based architectures due to security and privacy concerns, and external integration often requires a custom API for each system. Historically, web search engines were created to organize and index the content of the World Wide Web (WWW) [19], which was initially introduced as a collaborative platform for sharing human-readable documents.

While many IoT applications understandably demand strict privacy and security, the potential benefits of a collaborative framework for publicly accessible IoT data have been largely overlooked—especially when considering the transformative impact the WWW has had on human society. SensorsConnect [14] addresses this gap by providing a unified infrastructure for IoT systems, enabling the adoption of standardized protocols for sharing sensing data and laying the foundation for building an effective IoT search engine. The SensorsConnect framework is designed to support querying by both end users and integrated IoT systems.

#### SensorsConnect Architecture

**Figure 1:**
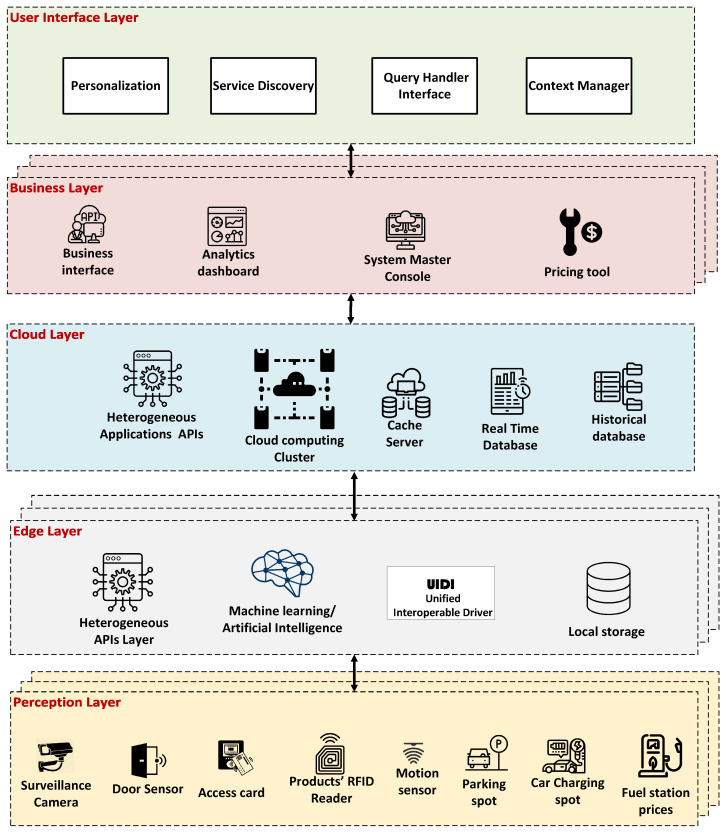
SensorsConnect Architecture [14].

Figure 1 illustrates the architecture of SensorsConnect, comprising five integrated layers: perception, edge, cloud, business, and user interface. The perception layer interfaces with the physical environment and manages the sensing devices. The edge computing layer functions as an adapter, converting heterogeneous sensing data into the Unified Interoperable Driver for IoT (UIDI) [20] format—an approach analogous to how HTML standardized content representation on the early World Wide Web. This standardization enables data sharing between IoT systems without requiring a custom driver for each integration. In certain scenarios, machine learning (ML) and artificial intelligence (AI) models are deployed locally at the edge layer handle data extraction tasks. For example, the edge layer can infer traffic volume from a surveillance camera at an intersection and transmit only the extracted data in UIDI format, rather than sending the entire video stream to the cloud layer [21]. The cloud layer manages IoT data through three main components:

1. A real-time database that stores the most recent updates from IoT devices.
2. A historical database that accumulates time-series data.
3. A cache server that retains frequently accessed data to reduce retrieval latency and optimize resource usage.

Additionally, SensorsConnect supports multiple cloud functions per IoT system. These functions allow systems to manage incoming data, interpret and enrich data with metadata (especially from resource-constrained devices), coordinate collaboration between IoT systems, and handle user queries effectively. The business layer provides enterprise-grade tools to manage IoT systems integrated into the framework. These include:

1. A master management console for device access across all layers,
2. An analytics dashboard for device monitoring and vulnerability detection,
3. A business interface for integrating third-party services (e.g., trade transactions or parking reservations), and
4. A pricing tool that suggests optimal pricing schemes based on business requirements.

The user interface layer includes components for query handling, service discovery [22], context management, and personalization [23]. These modules process user preferences, discover relevant services, extract contextual data, and tailor the experience to individual users. SensorsConnect operates via two primary workflows: Collect–Store and Query–Respond. The Collect–Store cycle governs the sensing, formatting, and storage of data—starting from the perception layer, passing through the edge layer, and concluding in the cloud’s data management components. In contrast, the Query–Respond cycle is activated when a user submits a query through the interface layer. The query is interpreted in the cloud layer and matched with relevant IoT data based on user intent. This paper focuses on the realization of the Query–Respond workflow. Since the data management component is shared between both workflows, this paper also introduces a unified data model designed to handle IoT data heterogeneity while supporting the requirements of both data collection and querying processes.

### 2.2. Related Work

The World Wide Web faced challenges in searching and organizing content in its infancy stages. Crawling, indexing, and ranking approaches [15] have been used to overcome searching for content in an unorganized world of content. Researchers in the natural language processing (NLP) field have recently presented methodologies to tackle searching tasks with techniques that achieve remarkable performance. The architecture of SensorsConnect [14] facilitates integrating search engines that leverage these approaches and overcoming limitations in traditional search methods.

#### 2.2.1. Large Language Model

Recently, there has been a major breakthrough in the field of Natural Language Processing (NLP), driven by the emergence of Large Language Models (LLMs) [17] built on transformer architectures [24] and trained on web-scale corpora. Traditional rule-based chatbots, such as Google Assistant, Siri, and Alexa, are limited to predefined user intents. When a user triggers one of these intents, the chatbot responds with a static, predefined answer that retrieves the relevant data. In contrast, LLM-powered applications like ChatGPT—based on GPT-3.5 or GPT-4 [25]—offer unprecedented capabilities, including:

1. Generating human-like responses that are not explicitly predefined,
2. Solving general-purpose tasks such as code generation,
3. Following user instructions for novel and complex tasks,
4. Executing multi-step logical reasoning when required, and
5. Learning from examples provided within the user prompt (few-shot learning).

As a result, LLMs have become a foundational technology for building generalpurpose AI agents. However, models like GPT-4 are not sufficient as stand-alone solutions for the search engine component of SensorsConnect, due to several inherent limitations:

1. **LLMs may hallucinate:** Trained on a mix of accurate and inaccurate data, they can generate confidently incorrect responses;
2. **LLMs are memoryless:** They lack persistent memory and cannot recall previous user inputs across prompts in a session;
3. **LLMs perform poorly on long-tail queries:** While effective on common knowledge, their accuracy diminishes for niche or specialized topics;
4. **LLMs cannot access real-time or external data:** They are trained on historical datasets and have no access to live sources, such as real-time data from IoT devices in SensorsConnect. Consequently, they are unaware of the current time or any data not present in their training corpus.

#### 2.2.2. Retrieval-Augmented Generation RAG

LLMs have achieved state-of-the-art performance across various NLP tasks due to their capacity to store vast amounts of knowledge within their parameters. However, as previously discussed, LLMs suffer from critical limitations: they are prone to hallucination, cannot retain or recall long-tail (uncommon) knowledge, and lack access to external or real-time data sources—such as the dynamic sensing data managed by SensorsConnect. To address these shortcomings, Lewis et al. [18] proposed the Retrieval-Augmented Generation (RAG) framework, which enhances LLM performance by incorporating a retrieval mechanism. RAG augments an LLM with access to an external database during inference time, allowing it to retrieve relevant information to support and enrich its generated responses. The RAG architecture consists of two primary components:

1. Retriever—identifies semantically relevant document chunks from an external database based on the user query.
2. Generator—combines the retrieved documents with the user input to produce accurate, contextually informed, and knowledge-grounded outputs.

This RAG approach [26] significantly improves the quality, coherence, and factual accuracy of LLM-generated responses. RAG techniques have been widely adopted in commercial systems, often referred to as “co-pilots” or LLM agents, which rely on RAG stacks to power domain-specific tasks. A recent survey [27] classifies RAG applications into eight categories: text, coding, knowledge, image, video, audio, 3D, and science. Nevertheless, these existing RAG implementations do not address the needs of search engines requiring access to live sensing data, as in the case of SensorsConnect. Most current RAG stacks rely on static embedding—where external data sources are ingested once and remain fixed. In contrast, the IoT-ASE use case demands continuously updated, real-time data, which imposes significant computational challenges if embedding were used. To resolve this, the proposed IoT-ASE system introduces a specialized IoT-RAG stack that accesses and processes real-time sensing data without embedding, enabling efficient, live-query support while minimizing computational overhead.

#### 2.2.3. Agentic LLM System

Multi-agent systems—also referred to as Agentic AI systems—have recently emerged as a promising paradigm for enhancing the capabilities of LLMs in solving complex tasks. Rather than relying on a single LLM agent to complete a task, multi-agent systems distribute responsibilities among multiple agents, enabling collaboration that improves task efficiency and reduces the likelihood of hallucinations.

The ReAct framework [28], a foundational approach in the development of agentic LLM systems, introduces a method that interleaves reasoning and acting in an iterative loop. In this architecture, the reasoning agent is responsible for generating, tracing, and refining action plans, while the acting agent executes these plans and interfaces with external data sources—such as knowledge graphs or simulation environments—to gather additional information.

Several domain-specific agentic systems have since been proposed in areas such as economics, social sciences, psychology, and healthcare. For example, CollabStory [29] is an agentic framework designed to collaboratively generate narrative content, while TradingGPT [30] engages in dialog and debate to inform decision-making in stock and fund trading, drawing insights from the ongoing discussion.

Despite these advancements, the field of Agentic AI remains in its early stages, with no universally accepted framework for designing optimal agent workflows across varying use cases. To address this gap, we introduce the GA-RAG workflow in this paper, offering a flexible architecture that can be adapted to a wide range of agentic systems.

#### 2.2.4. Sensors Data Retrieval Based on LLM

Dong et al. [31] introduce ChatIoT, a prototype system that integrates RAG to enhance IoT security incident response. The architecture comprises:

1. A retriever built on heterogeneous IoT security datasets (e.g., vulnerability reports, threat feeds), which are preprocessed into vectorized, document-style chunks.
2. An LLM (LLaMA3, LLaMA3.1, GPT 4o) layered atop this retriever, guided via domainspecific prompts and user context.
3. DataKit, a toolkit that automates parsing and optimal chunking of diverse data formats.

Empirical evaluation demonstrates that, when using LLaMA3-70B with RAG augmentation, ChatIoT achieved 10%+ improvements in relevance and technical depth over LLM-only baselines. This underscores RAG’s ability to ground generative models in up-to-date, high-fidelity IoT security intelligence, reducing hallucinations and enhancing trustworthiness. The framework proposed in [32] utilizes LLMs to enable sensor dataset retrieval from the web. It consists of two primary components:

1. A deployed LLM that extracts sensor data presented in HTML format on web pages and converts it into a structured format such as CSV.
2. A second LLM that generates word embedding representations of the extracted tabular data to support semantic search functionality.

The authors evaluated multiple LLM models using Precision at One (P@1) and Mean Reciprocal Rank (MRR) as performance metrics. Their results showed that the base-largeen-v1.5 model achieved the highest performance, with P@1 = 0.90 and MRR = 0.94, outperforming the other evaluated models. However, this framework does not support real-time data retrieval. The evaluation was conducted using historical data, with 90% of datasets stored as the target corpus and the remaining 10% used as input or query tables. Furthermore, as noted earlier, the framework uses LLMs to generate word embeddings for sensor data—which is typically numerical in nature. Since word embeddings are designed for textual data (words or sentences), applying them to numerical sensor readings is computationally inefficient and semantically imprecise. In addition, the author encountered several practical challenges, including dataset discovery, webpage heterogeneity, and API rate limitations, all of which affect the accuracy and consistency of the LLM used for extracting sensor data from web pages. To overcome these limitations, we propose the SensorsConnect framework, which introduces several key innovations:

1. A real-time search engine for sensor data retrieval,
2. A unified interface for IoT devices to mitigate heterogeneity, and
3. An efficient data management approach that allows seamless storage by IoT devices and real-time retrieval by LLM agents.

## 3. System Architecture

**Figure 2:**
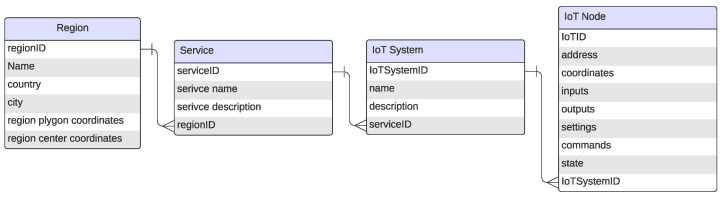
IoT-ASE Logical Data Model.

**Figure 3:**
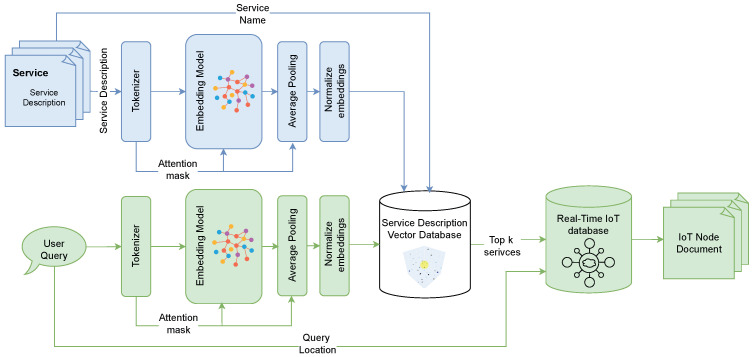
IoT Retrieval-Augmented Generation-Search Engine (IoT-RAG-SE).

**Figure 4:**
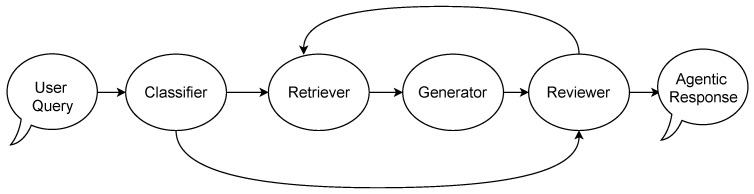
Generic Agentic RAG (GA-RAG) System State Diagram.

**Figure 5:**
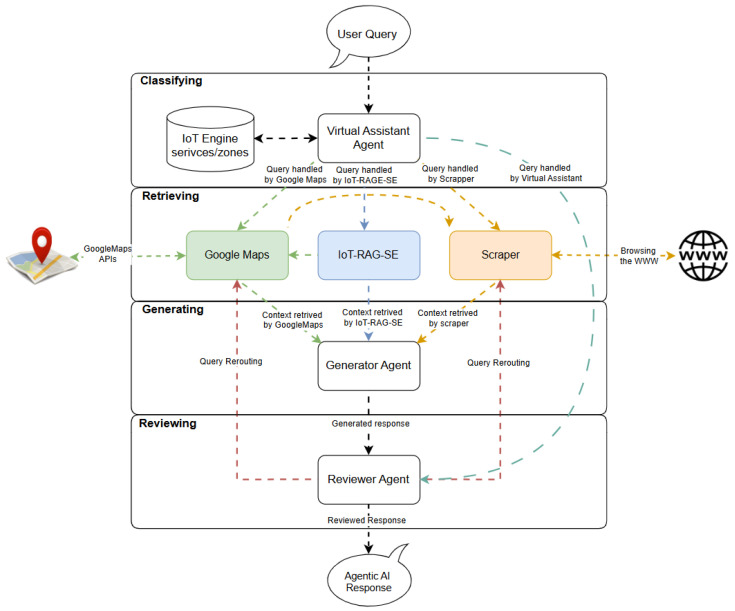
IoT Agentic Search Engine (IoT-ASE).

This section presents the key architectural components illustrated in the following figures:

1. The Unified Data Model Figure 2—designed to address the heterogeneity of IoT data.
2. IoT-RAG-SE Figure 3—responsible for processing IoT queries and retrieving real-time sensor data.
3. GA-RAG Workflow Figure 4—defines a systematic approach for constructing agentic RAG-based systems.
4. IoT-ASE Figure 5—an implementation of GA-RAG tailored for real-time IoT data environments.

Next, we explain each component in detail.

### 3.1. Data Model

Humans interact with web-generated content by leveraging their cognitive ability to interpret information, supported by advances in User Experience (UX) and User Interface (UI) design. Despite the diversity of website structures, content is rendered uniformly across devices with web browsers, thanks to standardized transfer protocols (e.g., HTTP) and data representation formats (HTML, CSS, and JavaScript).

In contrast, IoT data is fundamentally different in nature. A wide range of sensors and connected devices continuously generate real-time data, often represented as highdimensional time-series vectors. Although IoT systems commonly use communication protocols such as MQTT, CoAP, and HTTP, there is no universal standard for defining payload structures—unlike the consistent use of HTML in the World Wide Web. This absence of standardization presents significant challenges in processing and interpreting IoT data. To address this, SensorsConnect introduces a unified communication model by standardizing five core topics within the Unified Interoperable Driver for IoT (UIDI) messaging protocol:

1. Input—receives and processes sensor data,
2. Output—manages actuations or device responses,
3. Setting—configures operational parameters,
4. Command—enables external control over devices, and
5. State—communicates the device’s current status.

This streamlined structure plays a role similar to that of HTML in the WWW, offering a universal, lightweight protocol that promotes consistent development and seamless integration of IoT systems across diverse environments. We conducted an in-depth quantitative analysis [33] of the proposed data model’s effectiveness in managing heterogeneous IoT data. The evaluation included:

- Assessing the collaborative data-sharing needs of IoT devices, including those with limited computational resources, and
- Identifying the requirements of LLM agents, particularly their ability to query, interpret, and generate content based on structured IoT data.

Our findings demonstrate that the proposed IoT-ASE can adopt the document-based data model shown in Figure 2 to satisfy the operational needs of both IoT devices and LLM agents. Given the widespread presence of IoT devices in physical environments, most queries have a geographic component, making the management of large volumes of real-time data essential. To support this, we propose a hierarchical IoT data model, starting with the region entity for spatial partitioning. Each region organizes a set of services, which in turn includes multiple deployed IoT devices. At the lowest level, the node data entity encapsulates abstracted IoT data transmitted via the UIDI protocol, optimizing communication for resource-constrained devices. In parallel, the node entity provides rich descriptions and metadata, ensuring the data is machine-readable and interpretable by LLMs, thereby enhancing overall system intelligence, semantic understanding, and user-centric interaction.

### 3.2. IoT-Retrieval-Augmented Generation-Search Engine (IoT-RAG-SE)

IoT-RAG-SE processes heterogeneous, real-time IoT sensing data and routes user queries based on temporal and spatial parameters. Leveraging the Sentence-BERT [34] technique for sentence embeddings and a vector database built on the Hierarchical Navigable Small World (HNSW) algorithm [35], as illustrated in Figure 3, IoT-RAG-SE performs two core functions:

1. Embedding service descriptions into a high-dimensional vector space, and
2. Conducting semantic search to match user queries with relevant IoT services.

#### 3.2.1. Embedding Service Descriptions

To generate high-dimensional embeddings for each service description stored in the service entity defined in the data model (Figure 2), the input undergoes a structured sequence of processing steps, detailed as follows:

- **Tokenization:** A pre-trained tokenizer segments the service description into predefined tokens and produces both token IDs and an attention mask for each sentence.
- **Embedding:** Using the generated token IDs and attention mask, an embedding model encodes the sentence into dense vector representations that capture the semantic meaning of the input text.
- **Pooling:** The resulting token embeddings are passed through a mean pooling operation, which averages the token vectors while factoring in the attention mask. This process yields a single embedding vector that encapsulates the overall semantic content of the service description, weighted by the relevance of each token.
- **Normalization:** The resulting embedding vector is then normalized to ensure consistency across all service embeddings. This step is critical for enabling accurate similarity comparisons—particularly when using cosine similarity during the semantic search process.
- **Storage in Vector Database:** Finally, the normalized embedding vector, along with the corresponding service name, is stored and indexed in the vector database. This enables efficient retrieval through approximate nearest-neighbor search techniques.

When a new or updated service description becomes available, it can be seamlessly passed through the same embedding pipeline and stored in the vector-based service description database. This ensures that embeddings remain consistent across services and can be refreshed as descriptions evolve, thereby reducing the risk of data staleness and improving retrieval quality, even in cold-start scenarios.

#### 3.2.2. Performing Semantic Search

The semantic search process is initiated when IoT-RAG-SE receives a user query requesting a specific service or IoT data. The query is first passed through the same preprocessing pipeline as the service descriptions—comprising tokenization, embedding, pooling, and normalization—to generate a normalized embedding vector that captures the semantic meaning of the query.

Next, a Hierarchical Navigable Small World (HNSW) [35] semantic search is conducted within the vector database, comparing the query embedding with the stored service embeddings. Instead of retrieving a fixed number of results (top-p), the system applies a top-p probability-based threshold, selecting all services whose semantic similarity scores

fall within the top p-percentile of the distribution. This dynamic selection enables more contextually adaptive retrieval based on similarity confidence.

Finally, using the retrieved service names and the geographic location specified in the query, the real-time IoT database returns the most relevant and location-aware IoT data documents. These documents are structured to be readable and interpretable by LLMs, facilitating further reasoning and generation tasks.

### 3.3. Generic Agentic RAG (GA-RAG)

Figure 4 illustrates the GA-RAG workflow, presented as a state diagram that incorporates four agents: *Classifier*, *Retriever*, *Generator*, and *Reviewer*. The *Classifier* agent determines whether a query is relevant to the GA-RAG-supported services. The *Retriever* fetches relevant data—primarily containing information required to generate a response—from embedded sources within the RAG pipeline or from other external sources integrated into GA-RAG. The *Generator* uses both the retrieved context and the original query to produce a candidate response.

Finally, the *Reviewer* agent evaluates the generated response before returning it as the final output, aiming to minimize the risk of inaccuracy. If the response is deemed unsatisfactory or unreliable, the *Reviewer* may either reformulate the query to retrieve better context or instruct the *Retriever* to access alternative data sources.

Beyond their individual responsibilities, the four agents interact in a tightly coordinated loop. The *Classifier* ensures that only relevant queries trigger the RAG workflow, thereby reducing unnecessary computation and avoiding irrelevant retrievals. Once the *Retriever* collects candidate contexts, it passes them to the *Generator*, which synthesizes a draft response. The *Reviewer* then acts as a quality-control gate, creating a feedback loop: if the generated response is inadequate, the *Reviewer* can redirect the *Retriever* toward different sources or suggest rephrasing the query, effectively orchestrating iterative refinement. This interaction establishes a dynamic pipeline in which agents specialize yet collaborate to continuously improve the response quality.

The functional separation of these agents offers several advantages. First, it introduces modularity: each agent can be independently improved, replaced, or scaled without disrupting the entire system. Second, it increases robustness by incorporating explicit checkpoints for validation (via the *Reviewer*), which helps prevent error propagation from one stage to the next. Third, it supports adaptability: by allowing the *Reviewer* to loop back to earlier stages, the system can handle ambiguous or difficult queries more effectively. Finally, this separation enhances interpretability and maintainability, since the roles and

decision boundaries of each agent are clearly defined. Together, these benefits contribute to building a more reliable and trustworthy GA-RAG pipeline.

### 3.4. Implementation Details

We used LangGraph [36] to build the IoT-ASE shown in Figure 5, based on the GARAG workflow shown in Figure 4. To that end, we implemented four agents: classifier, retriever, generator, and reviewer. The next sections provide details about each agent.

#### 3.4.1. Classifier

A virtual assistant agent is deployed to classify user queries based on the semantic context of the conversation and activate the appropriate *Retriever* node corresponding to the query type. If the query falls within the capabilities of the LLM, the virtual assistant—functioning as an LLM agent—generates a response and forwards it to the Reviewer agent for validation.

In addition, the virtual assistant has access to the region entity defined in the data model Figure 2, allowing it to determine the geographic coverage of the *IoT-RAG-SE* system. This spatial awareness is incorporated into the classification logic, enabling the agent to decide whether to route the query to *IoT-RAG-SE* or an external service such as Google Maps, depending on whether the requested service falls within the SensorsConnect coverage zone.

#### 3.4.2. Retriever

The Retriever node consists of three specialized sub-nodes:

- *IoT-RAG-SE* Subnode: This component follows the architecture outlined in Figure 3 and accesses the Service and Node entities defined in the data model, Figure 2. It generates a vector database of service descriptions and returns the top-p nearest node documents that best match the user’s intent, providing relevant context to the *Generator* node. Additionally, *IoT-RAG-SE* integrates the OpenRouteService API [37] to calculate travel time and distance matrices between the user’s location and the locations referenced in the retrieved documents.
- *Google Maps* Subnode: This subnode interfaces with Google APIs, such as the Text Search API, and is used when a query requests a service not available within *IoTRAG-SE* or targets a region outside the SensorsConnect coverage area. It returns place-related documents containing the necessary details to formulate a meaningful response to the user’s query.
- **Scraper** Subnode: This component handles queries unrelated to IoT services and beyond the reasoning capabilities of the LLM, such as requests involving current events or news. It retrieves relevant documents by scraping online content using the Tavily API [38], supporting both the search and data extraction processes.

#### 3.4.3. Generator

The *Generator* node formulates the IoT-ASE response by combining the user query with the retrieved contextual information. To enhance the quality and relevance of the generated output, the *Generator* activates a specific prompt template tailored to the corresponding *Retriever* subnode—whether it be *IoT-RAG-SE*, *Google Maps*, or *Scraper*. This adaptive prompting strategy ensures that the response is semantically aligned with the nature and structure of the retrieved data.

#### 3.4.4. Reviewer

The *Reviewer* node examines both the user query and the generated response to ensure that the output accurately addresses the user’s intent. In the case of *IoT-RAGSE*, the quality of the generated response heavily depends on the accuracy of service descriptions. Inaccurate or incomplete descriptions can lead to irrelevant or incorrect retrieved contexts. The *Reviewer* can identify and flag such errors, particularly when they stem from misrepresented service metadata.

Additionally, users may submit complex, preference-specific queries, such as: “Find an uncrowded Italian restaurant with a discount on family orders and excellent real-time reviews.” In such cases, the *Reviewer* verifies whether the generated response addresses all specified criteria—e.g., cuisine type (Italian), crowd level (uncrowded), discount offering, and review quality.

When using the *Scraper* subnode, the Reviewer focuses on semantic coherence and relevance. For example, if a user asks for someone’s identity, the *Reviewer* checks whether the generated response includes a person’s name or relevant identifying information. If the response lacks essential content or appears inconsistent, the *Reviewer* may reroute the query to an alternative *Retriever* node, as illustrated in the flow diagram in Figure 5.

While the *Reviewer* currently has limited capabilities for deep factual verification, this component significantly reduces the impact of LLM hallucinations in the prototype version of IoT-ASE. Future enhancements may include the integration of a fact-checking module to further improve accuracy and reliability.

## 4. Scenario Analysis and Evaluation

IoT-ASE offers services to two primary entities:

1. End users, who access the system through the user interface layer to support real-time decision-making, and
2. Integrated IoT systems, which use the framework to enable inter-device collaboration.

This section focuses on the end-user scenario, using a dataset that represents realtime IoT sensing data. Despite Google Gemini [39] being a multimodal large language model (MLLM) capable of processing and understanding text, images, videos, and audio effectively, the structured and lightweight nature of IoT data—which is far less ambiguous than human-generated content—means that a less complex LLM may still be sufficient to achieve high performance. Moreover, extracting relevant content from human-generated web data requires significant computational resources and complex algorithms, especially for web scraping and semantic filtering. In contrast, IoT data hosted on SensorsConnect is well-structured and directly relevant, making it a more efficient and accurate source for context generation and query resolution. In this context, IoT-ASE demonstrates strong potential for powering intelligent virtual assistants capable of delivering substantial value to users. For example, Google Maps currently recommends places based on user location and ranks results by proximity or estimated travel time. While it can estimate occupancy levels, it does not consider total service time (service duration plus travel time)—a metric we previously demonstrated to be more effective in certain use cases [14]. In addition, many practical preferences are currently unsupported by mainstream recommendation systems. For example:

- A user may want to find a park where barbeque is permitted and an unbooked soccer field is available, or
- A restaurant owner may need to locate wholesale traders offering the lowest prices for a monthly stock order to reduce expenses.

Such queries go beyond basic keyword matching. They require:

- Extraction of user preferences from prompts, personalized profiles, or contextual history,
- Access to structured IoT data that reflects those preferences, and
- Reasoning over complex, multi-criteria constraints.

IoT-ASE meets these demands by enabling rich, preference-aware recommendations powered by real-time IoT data and LLM-based semantic understanding.

### 4.1. Datasets

Google Maps [40] contains data for over 200 million places, many of which can serve as proxies for IoT systems. In this study, we selected the Toronto region as the representative urban area for a hypothetical deployment of IoT-ASE. To simulate real-time IoT sensing data, we developed a custom scraping tool that crawls Google Maps and collects data from services in the Toronto area with an online presence.

**Figure 6:**
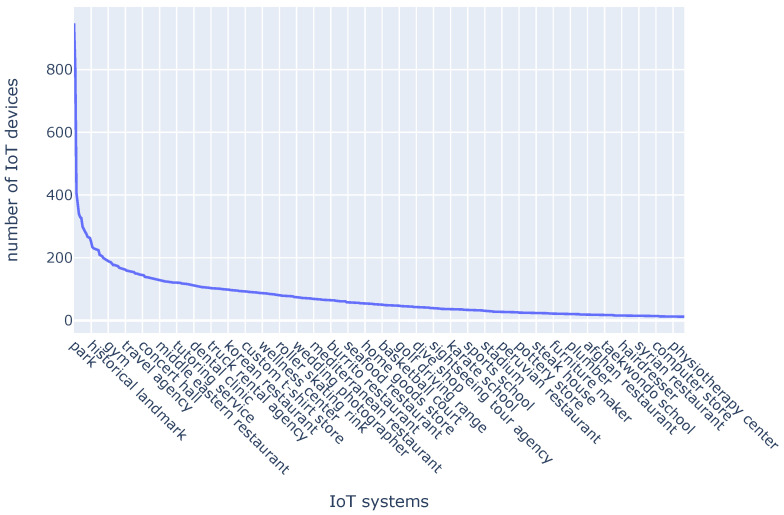
The distribution of IoT devices across a sample of services.

The resulting dataset includes 500 services, each considered as an IoT system integrated into the SensorsConnect framework. In total, we collected 37,033 instances, representing simulated IoT devices. Each service consists of multiple instances—for example, parks are grouped under the “park” service category, and each individual park is treated as an IoT device collecting real-time data. Figure 6 presents a line graph illustrating the distribution of IoT devices across a sample of services.

While the dataset is derived from Google Maps rather than physical IoT deployments, the simulated scenario closely mirrors real-world IoT environments in two ways: (i) the granularity of services and their instances approximates the heterogeneity of actual IoT deployments, where each entity (e.g., a park, hospital, or transit station) can host multiple sensors; and (ii) the spatial distribution across a metropolitan area reflects the geographic density and diversity typical of smart city infrastructures. Nevertheless, we acknowledge that this approximation does not fully capture the dynamics of real IoT systems—such as device-level failures, data quality issues, and communication overhead—which remain important aspects for future work.

The data cleaning process involved several steps to ensure accuracy, consistency, and usability. After scraping the raw data, we conducted validation to detect and handle missing values, duplicate entries, and formatting errors. In some cases, occupancy factor curves—which estimate the crowdedness of locations over their operating hours—were absent. Our analysis showed that places lacking occupancy curves were generally uncrowded, justifying the assignment of null values to their occupancy metrics.

Additionally, ratings were missing for some instances. Since services of the same type often exhibit similar patterns in user ratings, we used the average rating per service category as a substitute for the missing values—providing a statistically reasonable approximation for evaluation purposes.

### 4.2. Loading VectorDB and Real-Time IoT Database

We used the GPT-4 model [25] to generate service descriptions (Service descriptions: https://github.com/SensorsConnect/IoT-Agentic-Search-Engine/blob/main/src/vector_db/assets/Services_description_V2.txt, accessed on 5 September 2025) for the 500 services included in the case study. These descriptions were passed through the embedding pipeline and subsequently stored and indexed—along with their corresponding service names—in the service-description vector database.

In parallel, we stored the dataset of scraped services (places) in a MongoDB database, organized into 500 collections, each named after its corresponding service type as defined in the vector database. This design enables *IoT-RAG-SE* to route user queries based on both the requested service and the user’s location, using either 2D or geospatial indexing techniques. The system then returns the nearest matching places that align with the user’s intent.

**Figure 7:**
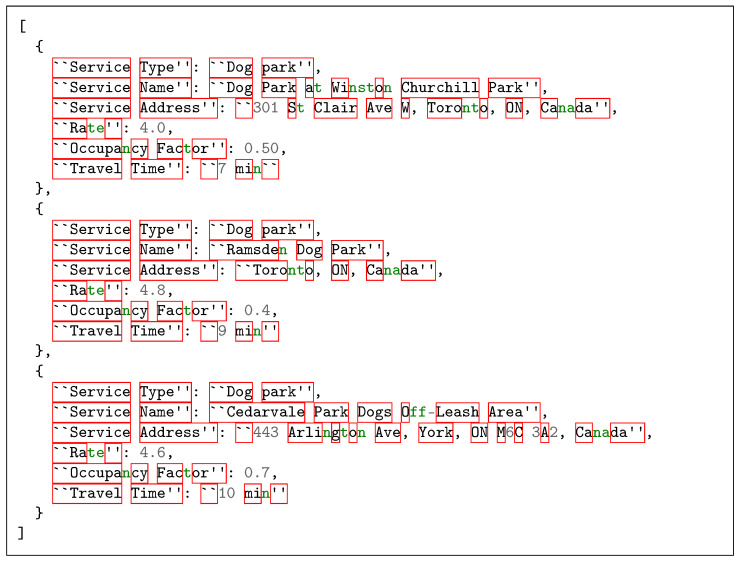
Example JSON Data for Dog Parks.

For example, consider the first query listed in Table 1: “I want to take my dog...” Upon receiving this query, *IoT-RAG-SE* returns the top-p matching service types—e.g., [“dog park”, “dog walker”, “dog trainer”]. The engine then uses these service names to identify the relevant MongoDB collections. Finally, leveraging geospatial indexing within each matched collection, the engine retrieves the nearest-place JSON documents, representing real-time IoT context data, as illustrated in the example output shown in Figure 7.

### 4.3. Evaluation

We evaluated the capabilities of IoT-ASE across three key dimensions:

1. Understanding complex queries that embed user preferences within contextual language,
2. Retrieving real-time IoT data documents necessary to identify the optimal service that aligns with user intent, and
3. Generating human-like responses based on the retrieved context.

The evaluation was performed using a curated set of 25 user queries, each targeting services with corresponding descriptions stored in the vector database and real-time instances stored in the IoT database. Unless a query explicitly specified a different location, the user’s location was assumed to be within the Toronto region. Queries referencing other cities were processed using the respective geographic context.

#### 4.3.1. Sematic-Search Evaluation

The *IoT-RAG-SE* component, illustrated in Figure 3, plays a critical role in the IoTASE workflow as a subnode agent responsible for query understanding and for enabling access to real-time contextual data. To evaluate its effectiveness, we conducted a series of experiments [33] comparing the performance of various data management systems for real-time IoT data retrieval.

Our findings indicate that the document-based data model outperforms alternative models in managing real-time IoT data—particularly in terms of latency across Create, Read, Update, and Delete (CRUD) operations and its flexibility in handling heterogeneous data structures.

To evaluate *IoT-RAG-SE*’s query understanding capability, Table 1 presents 25 user queries designed to assess its ability to infer the intended service from complex, preferencerich input. The queries were carefully selected to include relatively long contextual phrases, subtle semantic cues, and embedded user preferences, offering a robust test of the semantic search mechanism described in Section 3.

In this evaluation, the top-p parameter was set to 3, and the three most relevant service types retrieved for each query are listed in the Top-p Services column of Table 1. The results show that the correct intent was inferred within the top-3 results for all 25 queries. Notably, 92% of queries had the correct service ranked first, while for queries 9 (Tire Shop) and 10 (Museum), the intended service appeared as the third-ranked result.

Prioritizing the correct service as the top result is essential, as it allows the system to retrieve real-time IoT data directly from the corresponding service-specific collection, minimizing the amount of context passed to the LLM and thereby reducing token usage and computational cost. While retrieving documents from multiple service collections could help mitigate the risk of missing the correct service, such an approach is inefficient in terms of token consumption during LLM invocation.

This evaluation highlights the critical role of the quality of service descriptions stored in the vector database. Therefore, identifying strategies to improve, evaluate, and optimize these descriptions can significantly increase the likelihood of accurate first-result matches, enhancing both efficiency and relevance in the overall system response.

#### 4.3.2. IoT-ASE vs Gemini’s Responses

We highlight three sample queries to compare the quality and structure of responses generated by Gemini and IoT-ASE. The prototype (IoT-ASE Repository: https://github.com/SensorsConnect/IoT-Agentic-Search-Engine, accessed on 5 September 2025) (Live Demo: https://localelive.space/, accessed on 5 September 2025) used in this evaluation deployed the llama3-8b-8192 model [41] via the Groq API [42].

While Google Maps often displays 20+ location options, this breadth of information can hinder fast and effective decision-making. Similarly, Gemini’s responses—which rely heavily on Google Maps data via an integrated API tool—tend to include multiple options and generalized preference summaries, often listed in bullet-point format. In contrast, IoT-ASE is optimized to support real-time decision-making by generating concise, preference-aware recommendations. This is achieved by refining the system prompts to consider user preferences when weighing and selecting between possible alternatives.

**Figure 8:**
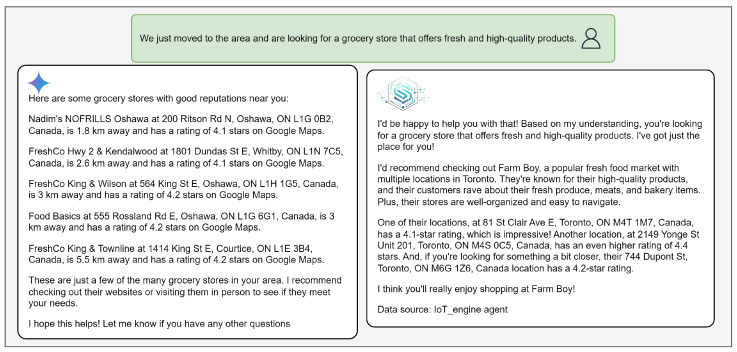
Comparison between IoT-ASE and Gemini responses to the Grocery store with a good reputation query.

**Figure 9:**
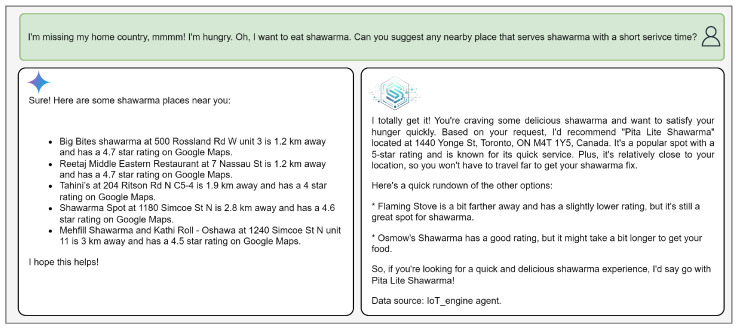
Comparison between IoT-ASE and Gemini responses to the Shawarma restaurant with a short service time supported only by IoT-ASE.

**Figure 10:**
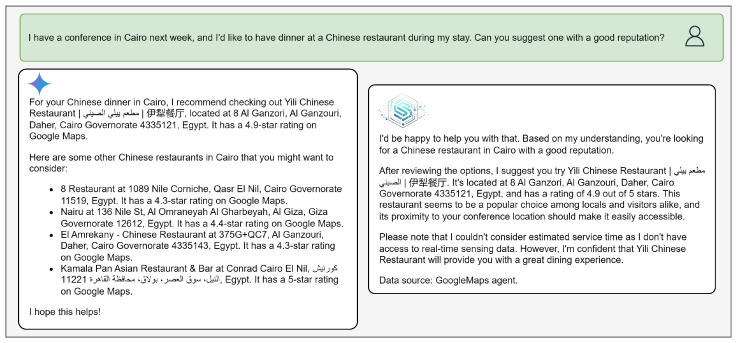
Comparison between IoT-ASE and Gemini responses to the Chinese restaurant in Cairo, highlighting recognizing the uncovered zone.

For example, in Figure 8, Figure 9 and Figure 10, Gemini responses resemble Google Maps results—structured as a list of suggestions without clear prioritization. IoT-ASE, by comparison, generates humanized responses that present a single recommendation, carefully selected after comparing three potential candidates based on the user’s implicit and explicit preferences. These examples consistently demonstrate that IoT-ASE provides more relevant, concise, and preference-aligned outputs. In contrast, Gemini responses often follow a generalized pattern and without ensuring that the listed options fully match the user’s intent.

In the grocery store query (Figure 8), the user seeks a reputable store. Gemini offers general suggestions, while IoT-ASE infers key elements—such as the service type, embedded preferences (e.g., reputation), and location. It then performs a semantic search to retrieve the top-p relevant services and uses real-time IoT data to rank the options. The generated response incorporates persuasive phrases like “fresh and high-quality products,” “a popular fresh market,” and “their customers rave about their fresh food.” Furthermore, it integrates travel time data from the OpenRouteService API, helping the user navigate to the nearest branch. In the shawarma query (Figure 9), Gemini lacks access to real-time occupancy or service-time data. In contrast, IoT-ASE retrieves real-time occupancy factors from the IoT database, combines them with travel time data, and produces a response tailored to minimize total service time—clearly reflecting user intent.

For the Chinese restaurant query (Figure 10), where the requested location is Cairo, a region outside the *IoT-RAG-SE* coverage zone, the *Virtual Assistant* agent (Figure 5) correctly detects this and routes the query to the Google Maps sub-agent. Even when relying on context retrieved from Google Maps, IoT-ASE produces a more conversational and useraligned response than Gemini. While it is acknowledged that Gemini outperforms IoT-ASE in general-purpose language tasks and broader prompt categories, this study demonstrates the practical applicability and architectural strength of IoT-ASE. Specifically, the system offers a way to connect LLMs to real-time IoT data sources, enabling faster, more accurate decision-making and avoiding the computational overhead associated with scraping and processing unstructured web content.

#### 4.3.3. Discussion

**Figure 11:**
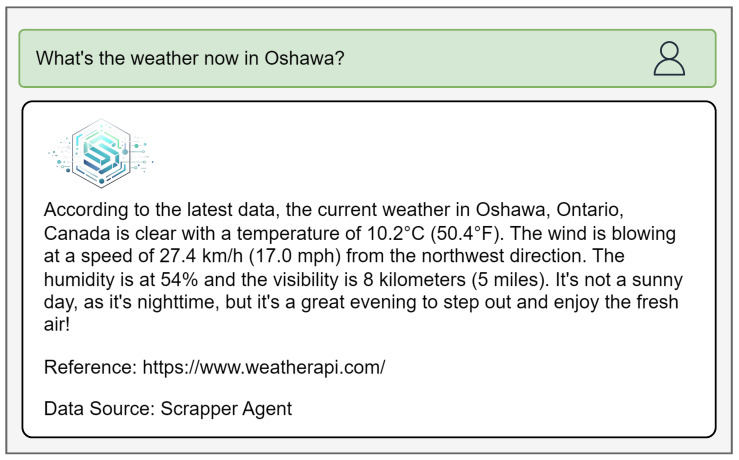
The response of IoT-ASE to the weather in Oshawa query by *scraping* agent that uses the weather API.

While LLM agents can rely on scraping methods to retrieve real-time IoT data for answering user queries, such approaches are computationally expensive and resourceintensive. For example, in Figure 11, a user requests the current weather conditions in Oshawa. Since IoT-ASE does not support a dedicated weather service, it reroutes the query to the Scraper agent, which utilizes the Tavily API to collect relevant context.

However, the retrieved result shown in Figure 11 was not obtained via scraping. Instead, it was sourced through WeatherAPI [43], an external API service that provides real-time weather data. This reveals that Tavily has begun integrating task-specific tools—in this case, a weather API—rather than performing generic scraping for such queries. While effective for certain domains, integrating an endless number of APIs for all IoT-related services is not a scalable solution. IoT-ASE addresses this challenge by using a unified data model (Figure 2), which eliminates the need for constant API integration or unreliable web scraping by enabling direct access to structured real-time data.

To assess the system’s performance, we used LangSmith [44] tracing tools to collect brief operational statistics. Over the course of 100 queries, IoT-ASE consumed 126,769 tokens, with a median of 1,330 tokens per query. The system demonstrated a low error rate of just 1%, representing unhandled queries due to internal processing failures. Additionally, 50% of the responses were generated in under 2.10 s, and 99% within 4.12 s. These metrics suggest that IoT-ASE is capable of delivering low-latency responses—critical for real-time applications—while maintaining high reliability.

**Figure 12:**
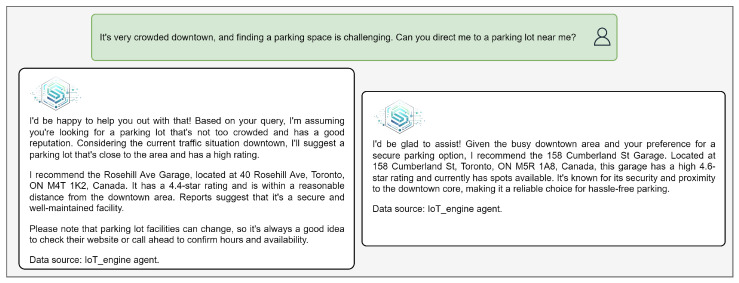
Comparison of IoT-ASE responses with and without the available parking spots field.

**Figure 13:**
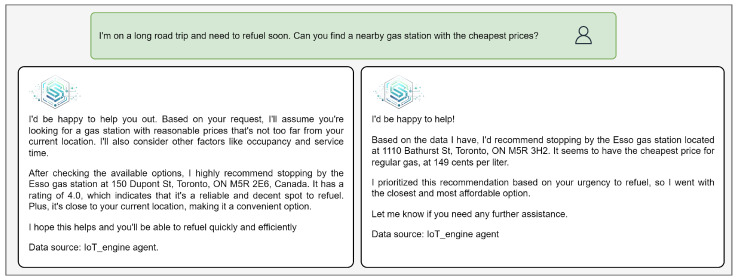
Comparison of IoT-ASE responses with and without the gas price field.

**Figure 14:**
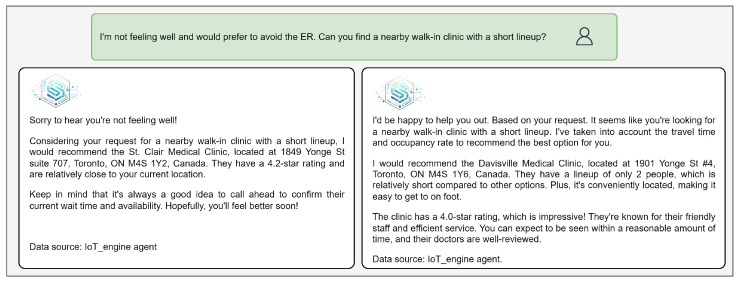
Comparison of IoT-ASE responses with and without the lineup counter field.

Figure 12, Figure 13 and Figure 14 illustrate real-world use-case scenarios that showcase the value of real-time IoT integration and the capabilities of IoT-ASE.

In Figure 12, a user located downtown during rush hour seeks an available parking spot. In the initial version of the documents used for context, the field indicating parking availability was absent. The Generator agent recommended an option based on other available metadata. To test the system’s ability to refine its recommendations, we updated the IoT documents to include a Boolean field indicating whether parking spots were available. After this update, IoT-ASE successfully selected a different location—one with confirmed availability—demonstrating how the system can adapt its reasoning based on live, structured data.

Similarly, in Figure 13, a user searches for a gas station with the lowest price. In the first run, without a gas price field in the documents, IoT-ASE generated a recommendation based on alternative factors such as distance, user ratings, and occupancy level, allowing the user to make an informed decision even in the absence of specific pricing data. After adding gas price fields to the documents, IoT-ASE was able to directly compare three fuel prices and recommend the cheapest nearby station, clearly aligning the output with user preferences.

In Figure 14, IoT-ASE addresses a query for a walk-in clinic with a short wait time. In the initial response—before integrating real-time lineup data—the system advised the user to call the clinic to confirm wait times. After adding a lineup field to each clinic’s document, IoT-ASE was able to automatically identify and recommend the clinic with the shortest current wait, helping to balance patient distribution and reduce overall waiting time. According to recent statistics [45], the average wait time at walk-in clinics in Toronto is approximately 72 min, making this optimization especially impactful.

Beyond these examples, numerous additional domains can benefit from real-time IoT integration. For instance, in industries dealing with perishable goods, such as the food sector, approximately 12% of retail food sales in Canada were classified as food waste in 2019 [46]. By connecting consumers with stores offering real-time discounts on near-expiration products, IoT-ASE can help reduce food waste while increasing business efficiency and customer satisfaction.

In summary, these case studies demonstrate that IoT-ASE not only improves real-time decision-making across diverse scenarios but also enhances the quality of life by enabling intelligent, data-driven recommendations grounded in live IoT data.

## 5. Limitations and Future Directions

While this paper contributes valuable concepts and efforts toward leveraging an IoT search engine, there are several limitations, and we must acknowledge them.

First, the datasets used in the case studies simulate real-time IoT data. We made efforts to obtain real-life scenarios by collecting real datasets from Google Maps in the Toronto area. The dataset was collected from Google Maps and stored in the MongoDB database. Hence, these datasets did not undergo continuous real-time updates, which introduces limitations regarding noise, diversity, and scalability compared to actual large-scale IoT environments. We consider the data stored in the conducted experiment to be the last update received from the integrated IoT devices, and the data were not continuously updated with the real status of services.

Second, the comparison between IoT-ASE and Gemini was made from our point of view and not from the feedback or input collected from participants who tried the search engine. We attempted to articulate hard-to-grasp queries containing relatively large contexts to validate the quality of responses generated by IoT-ASE. Additionally, some queries emphasized the impact of accessing real-time data on improving decision-making. Furthermore, evaluating *IoT-RAG-SE* includes a sample of 25 queries to evaluate the ability to infer the intended service implied in a complex query, which is considered a relatively small sample. Corporates with access to immense resources have the capacity to conduct the evaluation process with a comprehensive set of queries. For instance, we used 500 services with 37,033 instances in this experiment. An enterprise can articulate 10 queries, implying requesting each service, taking into account a wide range of customer profiles. Hence, 5000 queries can be sufficient to comprehensively assess the introduced IoT-ASE.

Third, while we conducted detached experiments for the crucial components, the end-to-end framework has not been tested on a large scale. For each step, we prioritized testing the main aspect that required checking before continuing in the same direction. We started the research seeking to build a search engine for IoT data. We couldn’t find well-known search engines serving the IoT data at this stage. Before starting to build the IoT search engine, we conducted motivating scenarios [14] (searching for a drive-thru coffee shop and crossing the US-Canada border) to estimate the potential impact of offering an IoT search engine. Then, after exploring existing data models that handle heterogeneous data, we came up with a hierarchically partitioned data model. We conducted a load test [33] for multiple implementations of the introduced data model using Apache Jmeter [47]. Lastly, our primary focus for IoT-ASE was to validate the ability to understand the users query and highlight the significance of reaching real-time IoT data while making decisions.

Although the paper validates IoT-ASE as a promising IoT search engine through the presented case study, sample responses, and similarity testing, many other aspects still require further evaluation. For instance, given that the framework will be accessed by millions of users, load and scalability tests are required to measure the framework performance under high traffic conditions and the capability of the systems to scale up and down based on the user traffic volume. Furthermore, the IoT-ASE requires reranking [48] and response accuracy testing, particularly when services significantly increase, and the current implementation hosts 500 services. Lastly, as the main objective of the framework is to enable real-time IoT data seeking to improve real-time decision-making, End-to-end latency is a crucial test. We have reported the average latency of the query-response cycle for IoT-ASE. However, these measurements were reported without considering high-volume requests.

Fourth, although our framework focuses on publicly accessible IoT data and therefore 736 does not directly process sensitive or private information, we acknowledge that security and 737 privacy remain critical considerations forbroader adoption. The current implementation 738 does not incorporate privacy-preserving retrieval mechanisms or secure query processing 739 techniques. Future work should extend IoT-ASE to address these aspects, for example 740 by integrating secure data access protocols, encryption schemes, or differential privacy 741 methods to ensure safe operation in contexts where sensitive IoT data is involved.

Finally, the research is still in the lab and has not been tested in real life. We did not provide the IoT community with APIs to integrate their systems into the framework. Moreover, we have not yet decided which incentive mechanism we will adopt in the framework to encourage IoT corporations and enterprise entities to share their public data. It’s crucial to see whether the community will embrace the idea or reject it. The success of this type of idea significantly depends on how widely it is adopted.

## 6. Conclusions

This paper proposes *IoT-RAG-SE*, a Retrieval-Augmented Generation (RAG)-based architecture designed to perform two primary tasks: (1) embedding service descriptions into a vector space, and (2) executing semantic search between user queries and service descriptions to retrieve relevant real-time IoT data from a dedicated IoT database—without embedding the IoT data itself.

Additionally, we introduce the GA-RAG workflow, derived from an analysis of existing agentic systems and a thorough evaluation of RAG architecture requirements. Building upon *GA-RAG*, we present the implementation of IoT-ASE, a complete system that operationalizes the workflow in real-time environments.

To validate the proposed architecture, we demonstrate a use-case scenario in which IoT-ASE functions as a virtual assistant that refines service recommendations by leveraging access to real-time IoT data. For experimentation, we hypothetically deployed IoT-ASE in the Toronto region using a dataset of 500 services simulating IoT systems and 37,033 JSON documents representing IoT device instances.

The evaluation consisted of 25 complex user queries, each embedding nuanced user preferences in natural language, designed to assess *IoT-RAG-SE*’s ability to accurately infer intent. The results indicate that *IoT-RAG-SE* identified the correct service as the top-ranked result in 92% of the cases.

We further conducted a qualitative comparison between IoT-ASE and Gemini using sample queries. Our findings show that IoT-ASE consistently generated more accurate, context-aware responses that addressed user preferences. In contrast, Gemini often produced generalized responses that lacked alignment with specific preferences embedded in the query.

While scraping publicly available web data or integrating numerous third-party APIs could, in theory, provide relevant IoT context for LLMs, these methods are computationally inefficient and difficult to scale. In contrast, SensorsConnect, together with the unified data model defined in this paper, offers a lightweight and structured solution for delivering relevant, real-time IoT context to LLM agents.

Based on our evaluation and discussion, we conclude that IoT-ASE is a promising and scalable solution for real-time IoT integration, enabling more intelligent, accurate, and preference-aligned decision-making in real-world applications.

## Figures and Tables

**Table 1:** The list of queries and their corresponding intents used in evaluating IoT-RAG-SE. Top-p services are the top P services names retrieved by IoT-RAG-SE.

#	Intent	Query	Top-P Services
1	Dog Park	I want to take my dog for walking and playing catch the ball, so I can unleash it	[’dog park’, ’dog walker’, ’dog trainer’]
2	Shawarma Restaurant	I’m missing my home country, mmmm! I’m hungry. Oh, I want to eat Shawarma. Can you suggest any nearby place that serves Shawarma?	[’shawarma restaurant’, ’middle eastern restaurant’, ’syrian restaurant’]
3	Moving and Storage Service	I’m planning to move to a new place. Do you know any moving agency close to me?	[’moving and storage service’, ’car rental agency’, ’travel agency’]
4	Gym	I have moved here recently, and I’m looking for a gym with a good reputation.	[’gym’, ’fitness center’, ’rock climbing gym’]
5	Car Rental Agency	I’m travelling tomorrow, and I want to rent a car. Do you know any car rental close to me?	[’car rental agency’, ’vehicle rental agency’, ’truck rental agency’]
6	Sports School	My son loves hockey sport, and I want him to start with professional practice playing it. Do you know any hockey school with a good reputation?	[’sports school’, ’hockey club’, ’ice skating rink’]
7	Zoo	My son loves animals, so I’d like to take him to a zoo	[”zoo’, ’wildlife park’, ’animal park”]
8	Chinese Restaurant	I have a conference at Toronto University next week, and I want to have dinner in a Chinese restaurant during my stay there. Can you suggest one with a good reputation?	[’chinese restaurant’, ’canadian restaurant’, ’chicken wings restaurant’]
9	Tire Shop	Winter is coming, and I need to install my winter tires. I’m looking for a place that offers discounts on this service.	[’auto parts store’, ’car detailing service’, ’tire shop’]
10	Gift Shop	My daughter’s birthday is next week. Can you suggest a store where I can have a variety of options for her gift?	[’gift shop’, ’toy store’, ’souvenir store’]
11	Cocktail Bar	It’s too hot, and I’m so thirsty. I really want to be hydrated with fresh juice. Do you have any suggestions?	[[’cocktail bar’ ’brunch restaurant’ , ’bar’,]]
12	Museum	I’m interested in learning more about the local history. Which museum do you recommend visiting?	[’memorial park’, ’tourist attraction’, ’museum’]
13	Hair Salon	I am planning to change my hairstyle and want to visit a top-notch hair salon. Can you suggest a hair salon with a good reputation?	[’hairdresser’, ’hair salon’, ’barber shop’]
14	Yoga Center	I’m stressed these days. Someone told me before that Yoga could help me relieve my stress. Do you have any recommendations for a Yoga center?	[’yoga center’, ’yoga instructor’, ’yoga studio’]
15	Furniture Store	We are redecorating our home and need to find a reliable furniture store with quality products. Can you recommend a furniture store with a good reputation?	[’antique furniture store’, ’furniture accessories’, ’home goods store’]
16	Dance School	My daughter loves dancing, and we are looking for a dance school where she can enhance her skills. Can you suggest a dance school with a good reputation?	[’dance school’, ’dance company’, ’music school’]
17	Martial Arts School	My son is very interested in learning self-defense, and we are looking for a reputable martial arts school. Do you know any martial arts school with a good reputation?	[’martial arts school’, ’karate school’, ’taekwondo school’]
18	Medical Spa	I am planning to treat myself to some relaxation and care, and I am looking for a medical spa with high standards. Do you know any medical spa with a good reputation?	[’medical spa’, ’massage spa’, ’massage therapist’]
19	Bakery	My daughter’s birthday is coming up, and she loves unique pastries. I’m looking for a bakery that can create a custom cake that’s both delicious and visually stunning.	[’bakery’, “children’s party service”, ’donut shop’]
20	Indian Restaurant	My family and I will visit the local area for a cultural festival. We’d love to experience authentic Indian cuisine while we’re there. Could you recommend one nearby with a good reputation?	[’indian restaurant’, ’modern indian restaurant’, ’middle eastern restaurant’]
21	Dentist	My daughter recently had a toothache, and we’re looking for a reliable dentist who is experienced with kids ages Could you recommend a good dentist nearby?	[’dentist’, ’dental clinic’, ’cosmetic dentist’]
22	Coffee Shop	I’m planning a casual meeting with a colleague next Tuesday morning. We’re looking for a quiet place to discuss some business ideas over coffee. Can you suggest a coffee shop that’s known for its serene environment?	[’coffee shop’, ’brunch restaurant’, ’lounge’]
23	Optometrist	My wife has been complaining about her vision while driving at night. We think it might be time for her to see an optometrist. Can you suggest a well-respected optometrist near us?	[’eye care center’, ’optometrist’, ’optician’]
24	Massage Therapist	I’ve been dealing with back pain due to long hours at my desk job. I heard that a good massage can help alleviate some of the pain. Do you know of a massage therapist nearby with excellent reviews?	[’massage therapist’, ’massage spa’, ’bank’]
25	Golf Club	It’s been a while since I didn’t enjoy playing golf. Do you know any nearby golf clubs with affordable membership subscription.	[’golf club’, ’golf shop ’, ’golf course’]

## Data Availability

The original data presented in the study are freely accessible at https://github.com/SensorsConnect/IoT-Agentic-Search-Engine, accessed on 5 September 2025.
